# Transcriptome analysis of the edible mushroom *Lentinula edodes* in response to blue light

**DOI:** 10.1371/journal.pone.0230680

**Published:** 2020-03-27

**Authors:** Jae Yoon Kim, Dae Yeon Kim, Youn-Jin Park, Myoung-Jun Jang

**Affiliations:** 1 Department of Plant Resources, College of Industrial Science, Kongju National University, Yesan, Republic of Korea; 2 Department of Biosystems and Biotechnology, Korea University, Seongbuk-Gu, Seoul, Republic of Korea; Ruhr-Universitat Bochum, GERMANY

## Abstract

*Lentinula edodes* is one of the most popular edible mushrooms worldwide and contains important medicinal components such as lentinan, ergosterol, and eritadenine. Mushroom metabolism is regulated by the mycelia and fruit body using light; however, in mushrooms, the underlying molecular mechanisms controlling this process as well as light-induced gene expression remain unclear. Therefore, in this study, we compared morphological changes and gene expression in the fruit bodies of *L*. *edodes* cultivated under blue light and continuous darkness. Our results showed that blue light primarily induced pileus growth (diameter and thickness) compared to dark cultivation. Alternatively, stipe length development was promoted by dark cultivation. We also performed RNAseq on *L*. *edodes* under the blue light/dark cultivation conditions. A total of 12,051 genes were used for aligning the Illumina raw reads and 762 genes that showed fold change cut-offs of >|2| and significance p-values of <0.05 were selected under blue light condition. Among the genes which showed two-fold changed genes, 221 were upregulated and 541 were downregulated. In order to identify blue light induced candidate genes, differentially expressed genes (DEGs) were selected according to 4-fold changes and validated by RT-PCR. We identified 8 upregulated genes under blue light condition, such as DDR48-heat shock protein, Fasciclin-domain-containing protein and carbohydrate esterase family 4 protein, FAD NAD-binding domain-containing protein that are involved in morphological development of primordium and embryonic muscle development, cell adhesion and affect the structure of cellulosic and non-cellulosic cell walls of fruit body development, and photoreceptor of blue light signaling for fruit body and pigment development, respectively. This study provides valuable insights into the molecular mechanisms underlying the role of blue light in mushroom growth and development and can thus contribute to breeding programs to improve mushroom cultivation.

## Introduction

Fungi belong to the eukaryotic group further including organisms such as yeast, mold, and mushrooms. Although fungi cannot photosynthesize due to their heterotrophic nature, generation of the mushroom fruit body appears to have a close relationship with light [1–3]. Recently, several studies have reported the environmental factors required for effective mushroom culture such as temperature and light [4,5]. Specifically, one report found that light induced browning led to mycelial formation in *L*. *edodes* [6].

Previous research found that light specifically plays a role in the effective regulation of secondary metabolites by the mycelia and fruit body of mushrooms [7]. For example, Sano et al. [8] reported that mycelia under light culture conditions showed more transcript accumulation than that of mycelia under continuous darkness. Additionally, regulation of the metabolic pathway in fungi was controlled by light [9]. Furthermore, in *Neurospora crassa*, light was involved in the pigment biosynthesis pathway in mycelia as well as in the progression of its reproductive stage [10,11]. These processes were ultimately caused by the white collar 1 (*wc-1*) and white collar 2 (*wc-2*) genes [12] which have been reported to act as photoreceptors and transcription factors and have moreover operated as a photomorphogenesis in *Agaricomycetes* [13,14].

Dark cultivation conditions also affect mushroom growth. For example, undesirable growth (e.g. cloudy mycelia color, reduced pileus growth) was observed in several mushrooms such as, *Grifola frondosa*, *Coprinus cinereus*, and *Hypsizygus marmoreus*, under dark culture conditions [15–19]. Shiratori et al. [19] further revealed that the color of mycelia was regulated by the amount and quality of light. Blue light is especially important for mycelia formation in several mushroom species [20]. Sano et al. [8] reported that blue light is required for fruit body development. Additionally, in *L*. *edodes*, pigmentation of primordia and development of fruit body were induced by blue light irradiation [21]. Furthermore, Sano et al. [8,22] identified photoreceptor genes from *L*. *edodes* including *phrA* and *phrB*. Katayose et al. [23] further identified the *pLLE1* gene, a linear mitochondrial DNA plasmid, and reported its putative role in photomorphogenesis of the fruit body caused by a photoreceptor.

There are several reports regarding blue light effects in mushrooms. Durand and Jacques [24] revealed photostimulation and photoinhibition in *Coprinus congregatus* operated at 445 nm. Additionally, Ellis et al. [25] reported that blue light is required for primordia formation and basidiocarp maturation in *Coprinus stercorarius*. Moreover, in *Flammulina velutipes*, pileus formation was regulated by photostimulation in accordance with light density, but stipe length decreased at 200 lux illumination [26]. Namba et al. [15] and Jang et al. [17] further reported that blue light at 460 nm and 475 nm, respectively, promoted pileus extension but inhibited stipe elongation in *Hypsizygus marmoreus*. Finally, mycelia growth in *Pleurotus ostreatus* was regulated by light intensity [3]. Alternatively, dark cultivation conditions induced primordia formation and stipe extension, and inhibited pileus formation in *Flammulina velutipes* fruit body [1].

Along with cultivation conditions and/or practices, genetic approaches are an effective alternative strategy to develop new mushroom cultivars or to ameliorate mushroom quality. Genetic improvement of edible mushrooms could be promoted by elucidating the molecular mechanisms underlying the fundamental processes occurring in these mushrooms such as photoreception, and by identifying various molecular markers and further understanding the target traits [27]. Recently, gene expression profiles of a target gene have been considered a powerful tool in mushroom breeding and could be useful in understanding physiological mechanisms. Song et al. [28] reported comparative transcriptome analysis between the mycelia and fruit body in *L*. *edodes*. These transcriptome data provide useful information regarding mushroom development.

In this study, we performed transcriptome analysis in *L*. *edodes* under blue light and dark cultivation conditions using Illumina sequencing technology. Identification of DEGs and assessment of their response to blue light will provide valuable information critical to further understand the molecular mechanisms underlying photoreception in *L*. *edodes*.

## Materials and methods

### Mushroom materials and culture conditions

The commercial *L*. *edodes* strain, Sanjo701ho (Accession no. ASI 3305), was developed by the National Institute of Crop Science (RDA, Republic of Korea). The medium to generate a mushroom fruit body was prepared in a sterile transplant plastic bag with oak tree sawdust (1120 g) and rice bran (280 g) with 58% moisture content. After sterilization at 121°C for 90 min, a spawn was inoculated onto the sawdust bag medium. For browning, the spawn was incubated at 21°C in the dark for 50 days and then exposed to light for 70 days.

In order to identify the expression profiles of the target genes in the developmental stage with or without blue light, the sawdust medium was transferred to the growth chamber under approximately 300 lux blue light. The cultivation conditions were maintained at 20°C and 80% humidity to generate a mature fruit body. Alternatively, the other *L*. *edodes* fruit body was incubated under the same cultivation conditions, but kept under continuous darkness and was used as a control mushroom. The pilei and stipes of the fruit bodies under both cultivation conditions were harvested at 2, 4, and 6 days (designated growth stage 1, 2, and 3, respectively) after incubation and stored at -80°C until further analysis. Student’s *t*-test was performed using SPSS 24 to determine if the means were significantly different at 5%, 1% and 0.1% probability level between blue light conditions or dark.

### RNA isolation

Total RNA extraction for library construction was prepared from whole fruit body using RibospinTM^II^ Kit (Geneall Biotechnology, Seoul, Korea) according to the manufacturer’s instructions. RNA quality was confirmed by the Agilent 2100 bioanalyzer using RNA 6000 Nanoship (Agilent Technologies, Amstelveen, Netherland). RNA quantification was determined using the ND-2000 spectrophotometer (Thermo Inc., DE, USA).

For gene expression profiles, total RNA was extracted from the pilei or stipes at the preferred growth stage in the same manner as described above. The RNA was analyzed by electrophoresis on 1% formaldehyde agarose gel (w/v), and then visualized by ethidium bromide staining.

### Library construction and Illumina sequencing

A total of 6 RNA-seq paired-end libraries were constructed from each 3 control (dark) and 3 blue light exposed pilei and fruit bodies at stage 3 (consisting of 3 biological replicates) using SMARTer Stranded RNA-Seq Kit (Clontech Laboratories Inc., CA, USA) following the manufacturer’s instructions. Two micrograms of total RNA from both the experimental and control fruit bodies were prepared for mRNA extraction using the Poly(A) RNA Selection Kit (LEXOGEN Inc., Vienna, Austria) and then used for cDNA synthesis and shearing procedures. Indexing was performed using Illumina index 1–12. The constructed libraries were later used for enrichment analysis using PCR amplification.

Each library was loaded onto the Agilent DNA High Sensitivity Kit (Agilent Technologies, Amstelveen, Netherland) to evaluate the mean fragment size. Subsequently, the libraries were used for quantification using a StepOne Real-Time PCR system (Applied Biosystem, CA, USA). High-throughput sequencing was performed to ensure attainment of the desired average sequencing depth using Illumina HiSeq 2500 (Illumina, CA, USA). After sequencing, the duplicated reads produced by PCR were filtered to obtain clean reads from raw data. The sequence data was trimmed using DynamicTrim and LengthScort, provided by SolexaGA [29].

### Short read mapping and functional analysis

The trimmed mRNA-Seq reads were mapped to Lentinedodes1 reference genome at JGI genome portal (https://genome.jgi.doe.gov/portal/pages/dynamicOrganismDownload.jsf?organism=Lentinedodes1) using the TopHat software tool with default option [30]. The raw RC (Read Count) were determined based on counts from unique and multiple alignments using coverage in Bedtools [31]. The RC -data were handled according to the quantile normalization method using EdgR within R (R development Core Team, 2016) and Bioconductor [32]. And, Each RC data was log2 transformed to compare and select differentially expressed genes (DEGs) for further functional analysis. Functional categories of putative upregulated genes (p-value < 0.05, log2 fold change > 2) were selected and blasted for comparing them to transcripts in the NCBI NR (non-redundant protein) database (e-value < 1e-5) [reference No.1], and classified by Blast2GO [reference NO. 2] with default option (annotation Cutoff = 55, GO weight = 5, E-value-hit-filter = 1.0E-6, Hit filter-500) to determine their molecular functions and biological processes. euKaryotic Orthologous Groups (KOG) of 2-fold upregulated genes were analyzed by the online KOG tool (WebMGA) [reference No.3] using blastx (e-value < 1e-3). Finally, KEGG (Kyoto Encyclopedia of Genes and Genomes) analyses were carried out using BlastKOALA [reference No.4] assigning KO identifiers to sequence data of 2-fold upregulated genes.

### DEG expression profiles and real-time quantitative PCR analysis

For qRT-PCR analysis, 1 μg of total RNA extracted from 2 different fruit body samples at stage 1, 2, and 3 was converted into first strand cDNA using a Power cDNA Synthesis Kit (iNtRON Biotechnology, Seoul, Korea) following the manufacturer’s instructions. The first strand synthesis was processed at 42°C for 60 min, followed by incubation at 95°C for 5 min to terminate cDNA synthesis reaction. The *L*. *edodes* 18S ribosomal protein (*18S rbs*), served as a housekeeping gene for normalization of the qRT-PCR data due to the expression stability of *18S rbs* in *L*. *edodes* [33]. The gene specific primer sets used for upregulation/downregulation of DEGs are listed in S4 Table. After diluting cDNA to acquire a 1/5 ratio, quantitative real-time PCR was performed with Rotor-Gene Q 2plex HRM (Qiagen, Hilden, Germany) using the Rotor-Gene SYBR Green PCR Kit (Qiagen, Hilden, Germany). The PCR reaction products (20 μl) were denatured at 95°C for 10 min, followed by 40 cycles of 10 s at 95°C, 15 s at an annealing temperature, and 1 min at 72°C. Amplification specificity was inspected by melt curve analysis from 65°C to 95°C. Three replicates of real-time PCR experiments were performed for each gene specific primer for all DEGs. The relative gene expression level was calculated using the 2^-ΔΔCt^ method and compared to the 1 day cultivated pileus sample under light conditions as a control [34].

## Results

### Morphological characterization of mushrooms under blue light and dark conditions

At stage 1 under blue light conditions, pileus diameter and thickness were 3.0 and 4.1 mm higher than the pileus of the fruit body under dark conditions, respectively, whereas stipe length and diameter were 10.3 and 0.2 mm smaller than the fruit body under dark conditions, respectively. At stage 2 under blue light conditions, pileus diameter and thickness were 18.9 and 3.4 mm higher than the pileus exposed to continuous darkness, respectively, whereas stipe length and diameter were 6.7 and 1.2 mm smaller than the stipe of the fruit body under dark conditions, respectively. At stage 3 under blue light conditions, diameter and thickness of the fruit body pileus were 16.8 and 2.0 mm higher than the pileus of the fruit body exposed to continuous darkness, respectively, whereas stipe length and diameter were 19.4 and 2.6 mm smaller than the stipe of the fruit body under dark conditions, respectively.

### Differentially Expressed Genes (DEGs) and functional analysis

After quality evaluation and trimming, over 21.2 million trimmed reads and 2.2 billion bases on average were generated from each sample under dark conditions (no light, NL) and blue light (BL) conditions. Additionally, over 70% of sequenced data (average mapping rate: 72.86%) mapped to assemble transcripts from the NCBI database (Table 1). Among them, a total of 12,051 *L*. *edodes* genes were used for aligning the Illumina raw reads, and a total of 762 genes showed two-fold cut-off on fold changes (FC) under BL conditions. Of these genes, 221 (29%) were significantly upregulated (S1 Table) and 541 (71%) were downregulated under BL conditions. We focused on the 221 upregulated genes for functional analysis to evaluate the effect of blue light on molecular and phenotypic characteristics of *L*. *edodes*.

**Table 1:** The reads, bases number and mapping rate of the *L*. *edodes* transcriptome.

Sample	Reads	Trimmed reads	Bases No	Mapping rate (%)
blue light_1	21624698	20854456	2184094498	70.77
blue light_2	21247312	20288086	2145978512	72.71
blue light_3	17941476	17143198	1812089076	71.57
dark_1	22219996	21403894	2244219596	75.46
dark_2	25827588	24717108	2608586388	72.79
dark_3	23559370	22704002	2379496370	73.85
Average	22070073	21185124	2229077406	72.86

**Fig 1:**
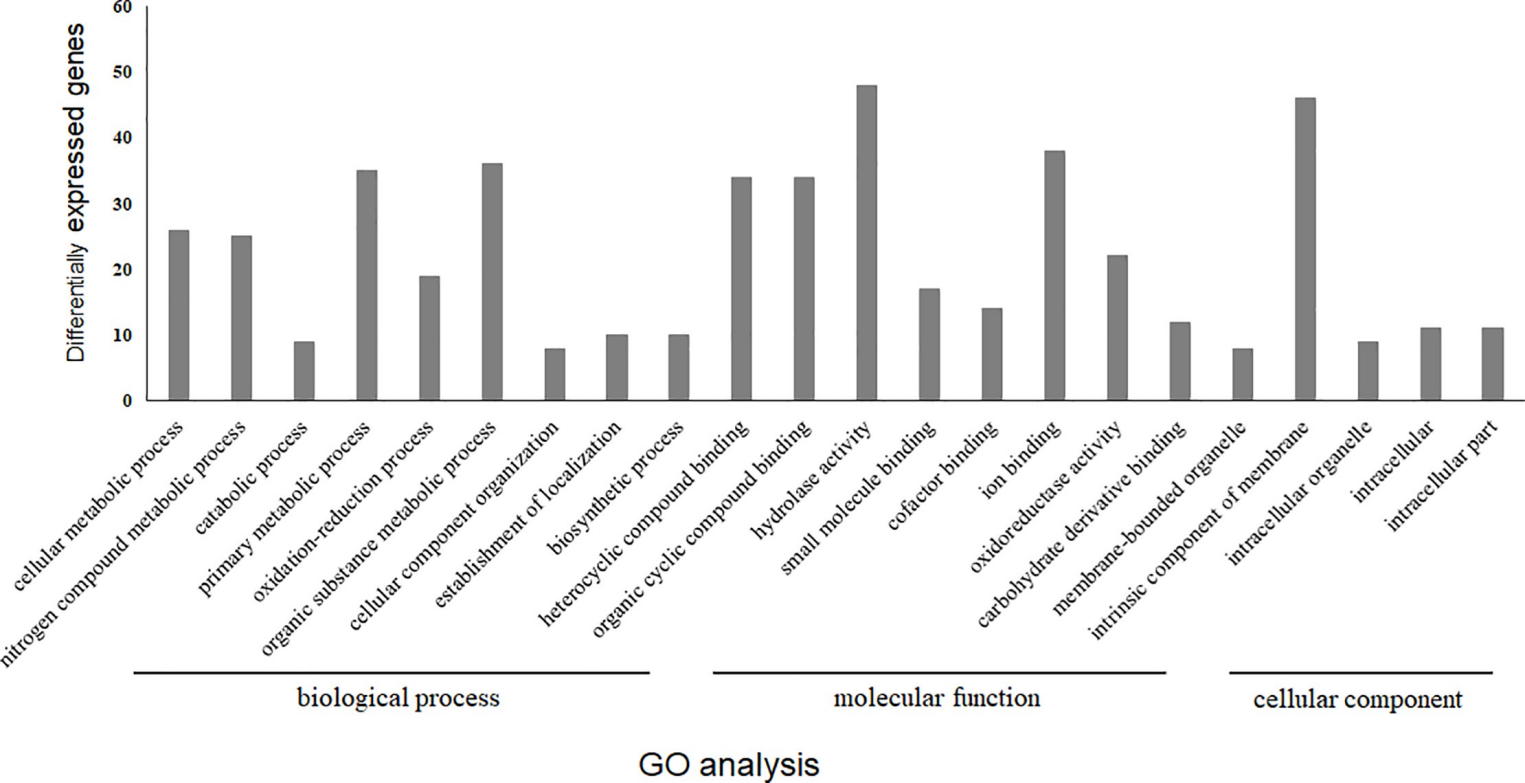
Gene ontology analysis of 2-fold upregulated genes under blue light conditions. GO annotation was conducted using the Blast2GO software and the genes were grouped into three main GO categories including, biological process, molecular function, and cellular component.

To investigate the function of upregulated genes, the identified genes were annotated by GO, KOG, and KEGG databases. For GO enrichment analysis, the 221 upregulated genes were analyzed using Blast2GO and we performed a local BLAST search of these genes using the proteins of *Basidiomycota* listed in the NCBI non-redundant (NR) database. Of the 221 upregulated genes, 150 were mapped to one or more GO terms; moreover, 178, 219, and 85 GO terms were mapped to biological process, molecular function, and cellular component categories, respectively (Fig 1 and S2 Table). GO enrichment analysis revealed that the biological processes, ‘organic substance metabolic process’ (GO:0071704, 36 genes) and ‘primary metabolic process’ (GO:0044238, 35 genes), were abundantly enriched under BL conditions. In the molecular function category, the GO terms ‘hydrolase activity’ (GO:0016787, 48 genes) and ‘ion binding’ (GO:0043167, 38 genes) were also abundantly enriched under BL conditions. Lastly, ‘intrinsic component of membrane’ (GO:0031224, 46 genes) was the most enriched category of cellular component under BL conditions.

**Fig 2:**
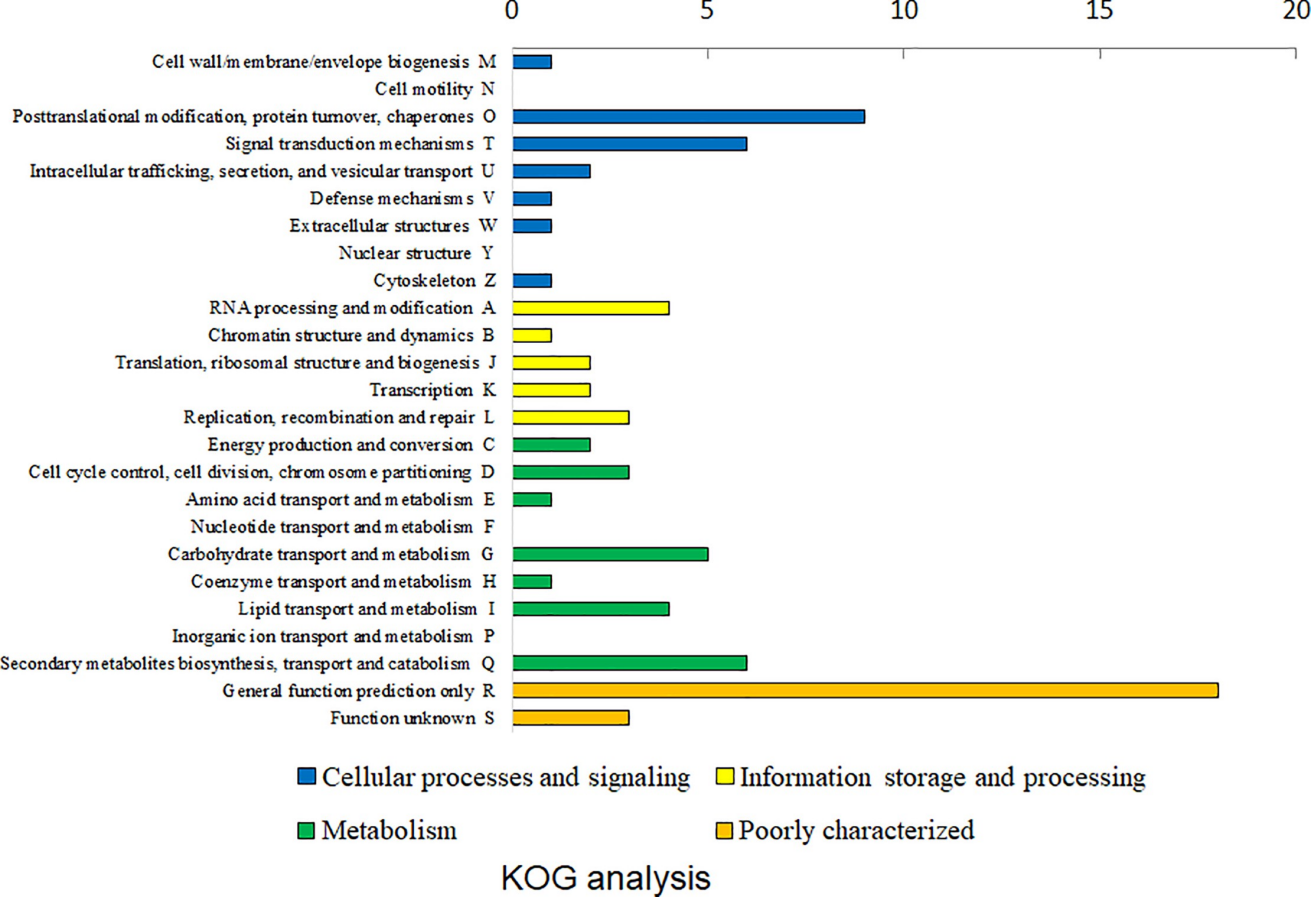
KOG functional analysis of 2-fold upregulated genes under blue light conditions. KOG analysis was conducted using the KOG online tool and the genes were sub-grouped into cellular process and signaling, information storage and processing, metabolism, and poorly characterized genes.

Enrichment analysis of KOG functional categories was conducted for detailed classification of upregulated genes under BL conditions (Fig 2 and S3 Table). The two terms, ‘Posttranslational modification, protein turnover, chaperones’ and ‘Signal transduction mechanisms,’ showed a high ratio in the cellular processes and signaling category; additionally, ‘RNA processing and modification’ and ‘Replication, recombination and repair’ highly matched with upregulated genes in the information storage and processing category. Lastly, within the metabolism category, ‘Secondary metabolites biosynthesis, transport and catabolism’ and ‘Carbohydrate transport and metabolism’ highly matched with upregulated genes under BL conditions.

**Fig 3:**
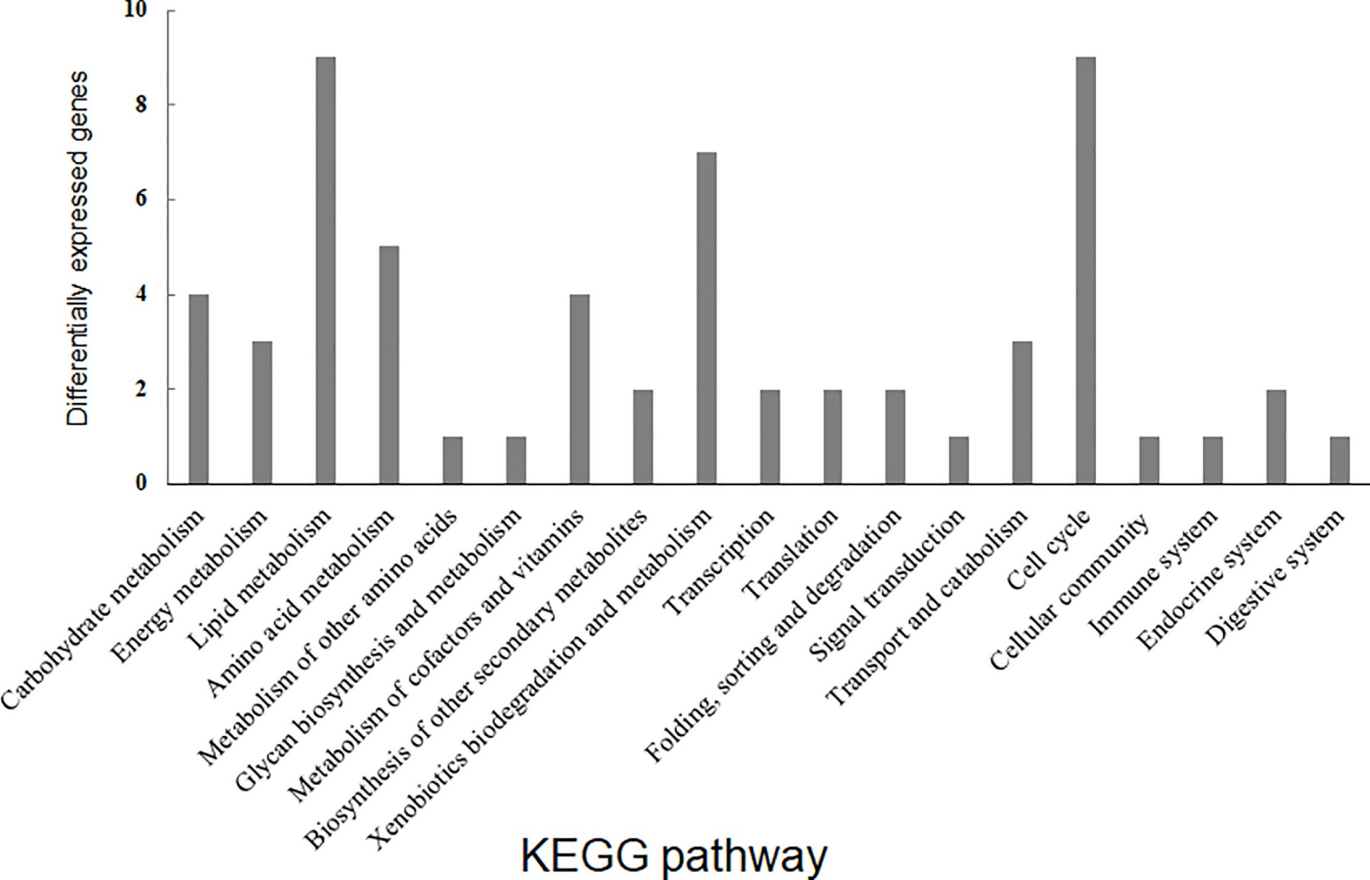
KEGG analysis of 2-fold upregulated genes under blue light conditions.

KEGG sub-classification was conducted to further evaluate the functional pathway derived under BL conditions. Among the 19 KEGG sub-classifications, the top three were ‘Lipid metabolism,’ ‘Cell cycle,’ and ‘Xenobiotics biodegradation and metabolism’ (Fig 3).

### Expression profiles of DEGs in the different developmental stages of *L*. *edodes* exposed to blue light

**Fig 4:**
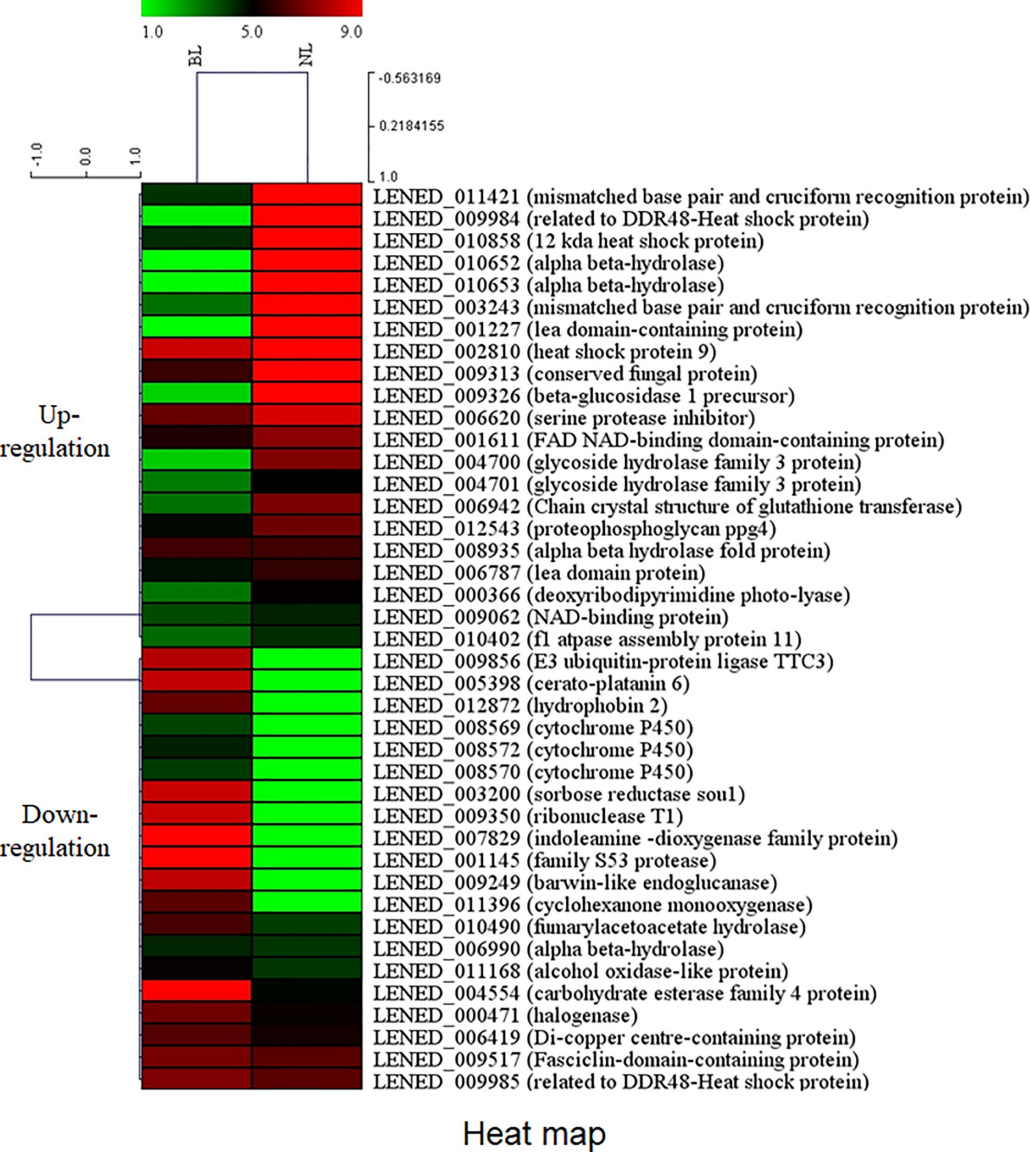
Heat map and hierarchical clustering analysis of 4-fold up- and down-regulated genes under blue light and dark conditions. Each column represents an experimental sample (no light and blue light), and each row represents a gene. Red and green colors indicate high and low expression of each gene, respectively. Each value on the heat map represents normalized reads count (RC) data using log2 transformation.

**Fig 5:**
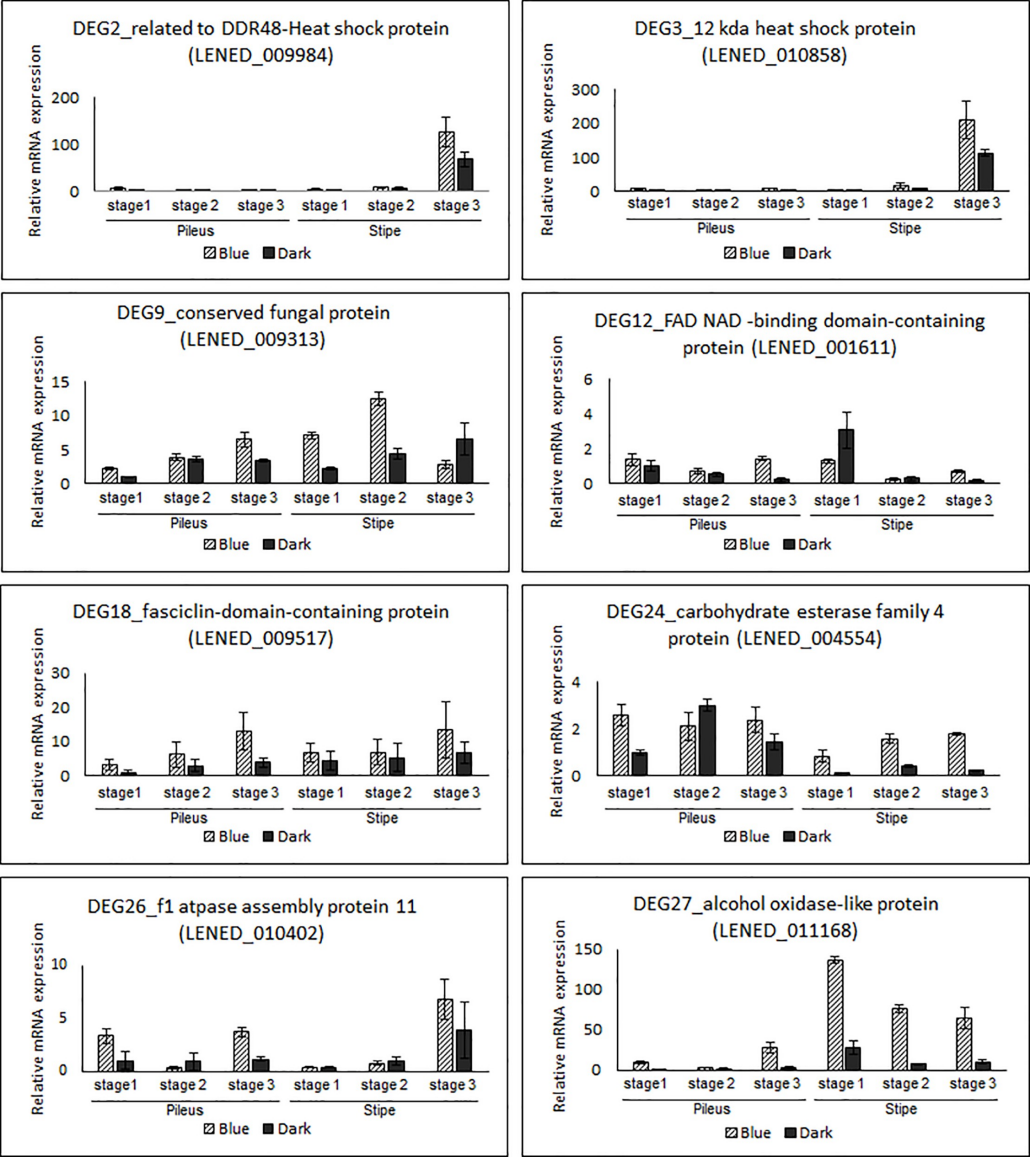
Validation of 8 upregulated DEGs according to a 4-fold change using quantitative RT-PCR. Each experiment was normalized by a housekeeping gene (*Rpl4*).

**Fig 6:**
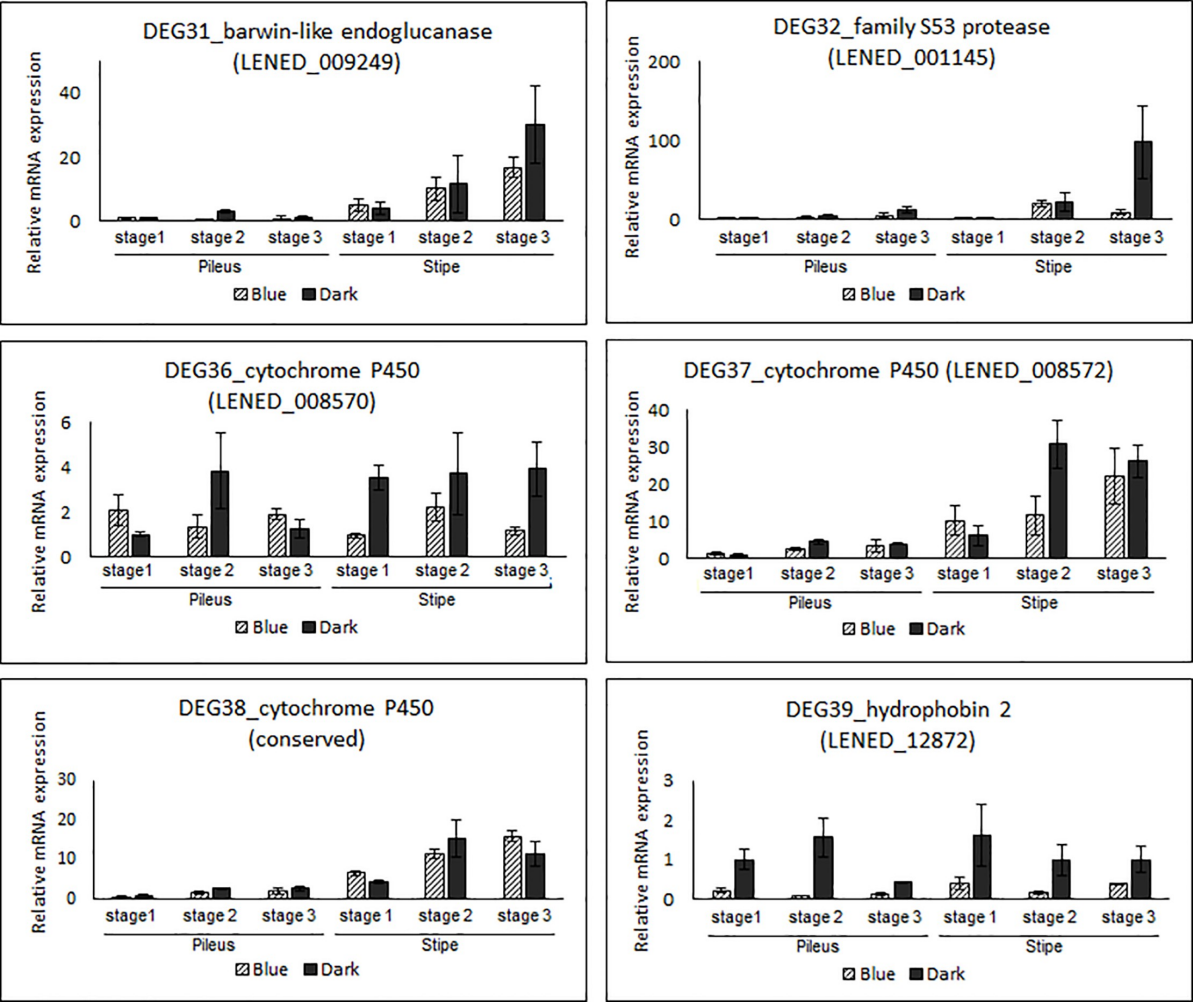
Validation of 4 downregulated DEGs according to a 4-fold change using quantitative RT-PCR. DEG39-1 and DEG39-2 represent the same cytochrome P450 gene but a different sequence. DEG39-3 represents qRT-PCR results performed with cytochrome P450 specific primers which were designed at conserved sequence regions. Each experiment was normalized by the *18S* housekeeping gene.

To narrow down the number of target DEGs under blue light, we selected 41 DEGs (26 upregulated and 15 downregulated) under BL conditions according to 4-fold changes. The hierarchical clustering and visualization analysis of up- and down-regulated genes were shown in Fig 4. After qRT-PCR, stable and reproductive PCR was performed using 23 DEGs (15 upregulated and 8 downregulated). Eventually, 12 DEGs (8 upregulated and 4 downregulated) corresponded with RNA sequencing results (Table 2). Of the upregulated DEGs, DDR48-heat shock protein, 12 kDa heat shock protein, and alcohol oxidase-like protein were observed at very high expression levels in the stipe tissue of *L*. *edodes* (Fig 5). Interestingly, the expression of DDR48-heat shock protein and 12 kDa heat shock protein increased with mushroom growth, whereas alcohol oxidase-like protein expression decreased. FAD NAD-binding domain-containing protein, fasciclin-domain-containing protein, conserved fungal protein, carbohydrate esterase family 4 protein, and f1 ATPase assembly protein 11 were dominantly expressed in both the pileus and stipe tissues of *L*. *edodes*. Interestingly, the transcripts of fasciclin-domain-containing protein, conserved fungal protein, and carbohydrate esterase family 4 protein increased with mushroom growth. Additionally, conserved fungal protein expression rapidly decreased in the stipe tissue at growth stage 3. Moreover, transcripts from the stipe of dark-exposed mushrooms showed higher expression level than those of blue light exposed mushrooms. Regarding downregulated expression of DEGs (Fig 6), barwin-like endoglucanase, family S53 protease and cytochrome P450 (LENED_0008570) expression increased with mushroom growth. Additionally, expression of these genes in stipe samples was higher than expression in pileus samples. Lastly, hydrophobin2 and cytochrome P450 (LENED_0008572) maintained high expression during all growth stages in both the pileus and the stipe tissues.

**Table 2:** Summary of blue light induced 4-fold change DEGs.

ID	Gene symbol	Log2 fold change^a^	Description	e-value	Identity(%)	expression
DEG2	LENED_009984	29.222	Related to DDR48-Heat shock protein	2E-94	100	up
DEG3	LENED_010858	26.880	12kDa heat shock protein	4E-50	100	
DEG9	LENED_009313	12.562	Conserved fungal protein	0.00	100	
DEG12	LENED_001611	7.206	FAD NAD-binding domain-containing protein	0.0	100	
DEG18	LENED_009517	6.408	Fasciclin-domain-containing protein	2E-166	100	
DEG24	LENED_004554	4.920	Carbohydratae esterase family4 protein	0.0	100	
DEG26	LENED_010402	4.299	F1 atpase assembly protein11	3E-65	100	
DEG27	LENED_011168	4.181	Alcohol oxidase-like protein	0.0	100	
DEG31	LENED_009249	0.215	Barwin-like endoglucanase	8E-100	100	down
DEG32	LENED_001145	0.208	Family S53 protease	0.0	100	
DEG36	LENED_008570	0.154	Cytochrome P450	0.0	100	
DEG37	LENED_008572	0.154	Cytochrome P450	0.0	100	
DEG39	LENED_012872	0.146	Hydrophobin2	3E-58	100	

^a^ indicates a significant difference between blue light cultivation and dark (no light) cultivation.

## Discussion

*L*. *edodes* is one of the most popular edible mushrooms worldwide and the study of its cultivation conditions is critical to enhance mushroom quality and economic market value [35]. Additionally, during fruit body development without low-temperature treatment, the expression of growth regulation genes was enhanced by light in *L*. *edodes* [36].

Several studies have investigated the role of light in the regulation of biological processes in fungi. For example, nucleoside diphosphate kinase (NDK) in *Schizosaccharomyces pombe* is involved in the regulation of various signal transductions such as sexual development and photomorphogenesis [37]. Additionally, several open reading frames (ORFs) of blue light receptors have been reported including, *dst1* from *C*. *cinereus*, and *wc-1* and *wc-2* from *Schizophyllum commune* and *Neurospora crassa* [8,16,22,38]. Tang et al. [39] further revealed that blue light was involved in brown film formation. Moreover, 73 protein spots were observed in blue light cultivated mushrooms using two-dimensional electrophoresis (2DE) mapping, which was twice the density observed in dark cultivated mushrooms [6].

**Fig 7:**
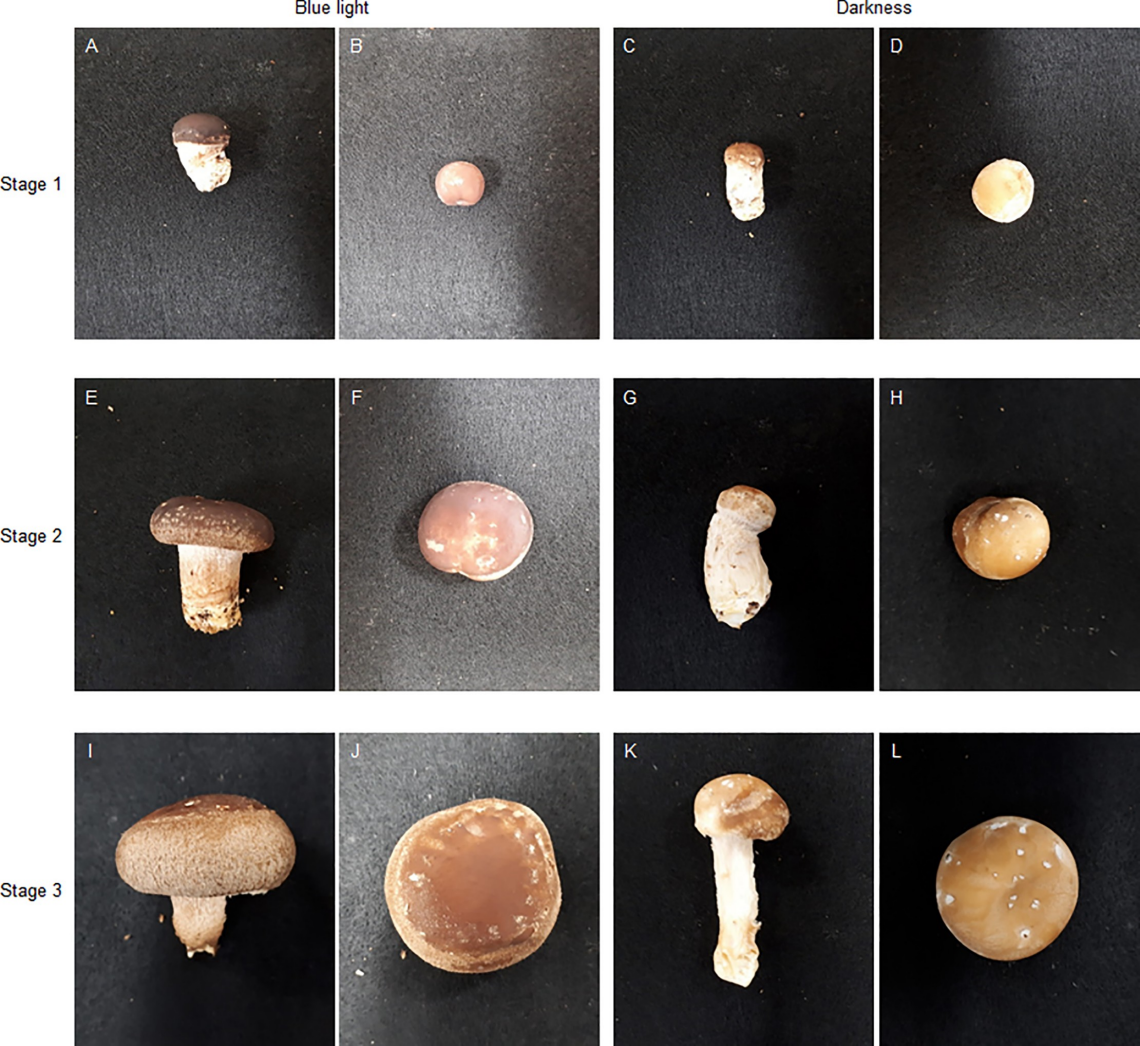
Morphological transformations according to different cultivation conditions. A, B, E, F, I, and J were cultivated under blue light. C, D, G, H, K, L were cultivated under darkness (no light). Growth stage 1, 2, and 3 represents harvest times (2, 4, and 6 days after beginning either treatment).

**Fig 8:**
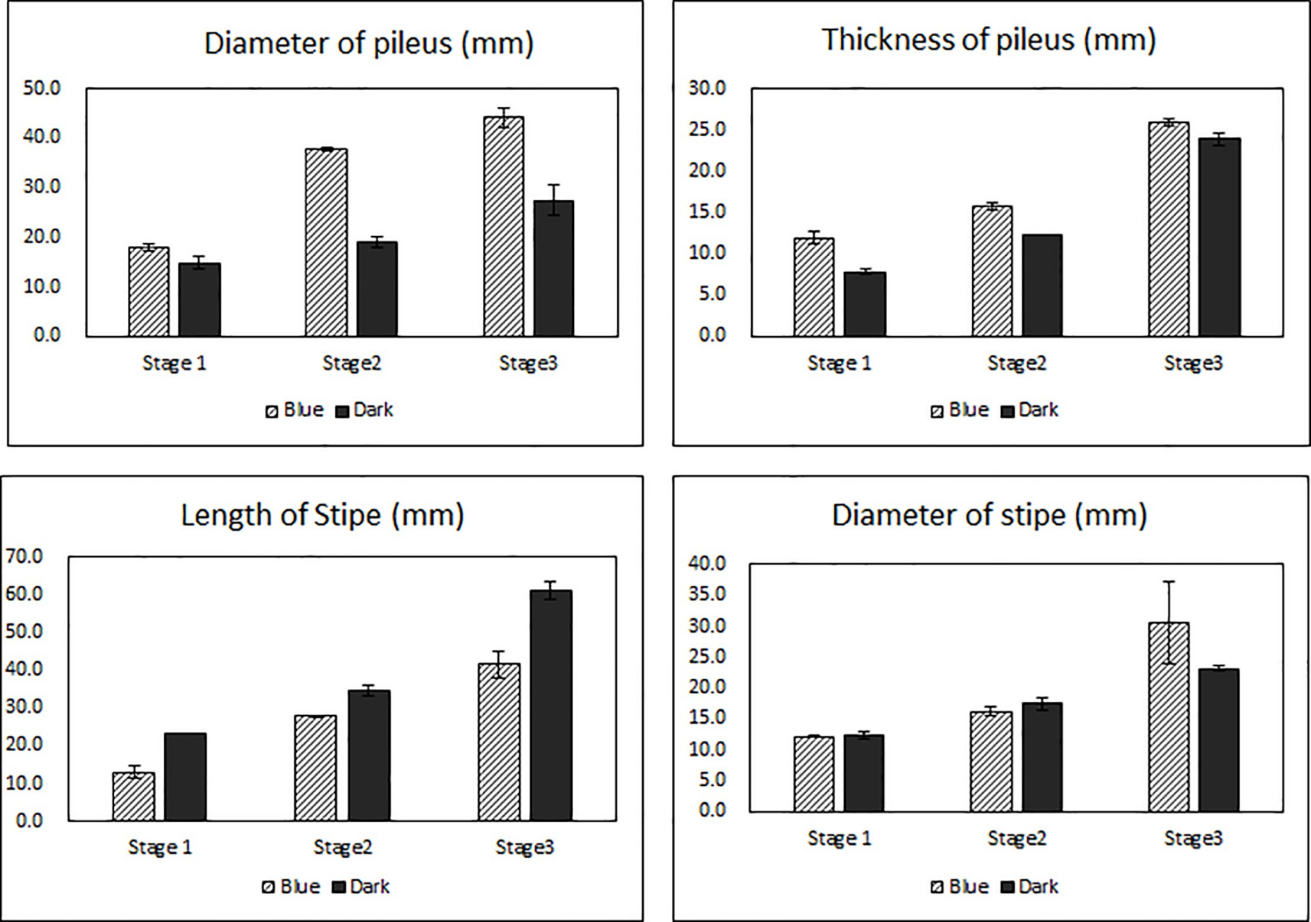
Comparison of pileus diameter and length as well as stipe diameter and length under blue light or dark conditions during fruit development. The black lines indicate diameter or length under blue light cultivation. Gray dashed lines indicate diameter or length under dark (no light) cultivation. Growth stage 1, 2, and 3 represents harvest times (2, 4, and 6 days after beginning either treatment).

Light can stimulate or inhibit fungi growth in accordance with developmental stage [40,41]. In mushrooms, blue light is considered an important environmental factor for fruit body development [20], and blue light receptor proteins were identified in *C*. *cinereus* and *L*. *edodes* [16,22]. As shown in Fig 7 and Fig 8, throughout fruit body development, the diameter and thickness of *L*. *edodes* pileus under blue light culture conditions was larger than the pileus under dark conditions; alternatively, *L*. *edodes* stipe length and diameter was smaller under blue light conditions than under dark conditions. Additionally, under blue light conditions, pileus color was darker and richer than pileus color under dark conditions. According to Leatham and Stahmann [21], blue light induced pigment formation during primordia development in *L*. *edodes*. Namba et al. [15] further reported that the pileus of *H*. *marmoreus* was larger in size and darker in color in mushrooms exposed to light compared with mushrooms exposed to continuous darkness during fruit body development. Sakamoto et al. [1] also reported that in *F*. *velutipes*, pileus diameter increased and stipe length decreased under blue light, whereas the opposite occurred under red light or darkness. These results correspond with the results of the present study (Figs 7 and 8). In this study, we further identified 8 upregulated and 4 downregulated DEGs under blue light conditions using RT-PCR. Of the identified upregulated DEGs, 2 were heat shock proteins. DDR48-heat shock protein was reported in abundance in the primordium (dikaryotic mycelium) of *L*. *edodes* [42] and 12 kD heat shock protein was involved in morphological development during the embryonic muscle development [43]. Because heat shock protein repress the denaturation of molecules by stressful environment [43], the DDR48-heat shock protein and 12 kD heat shock protein can be involved in important roles such as chaperone function in fruit body development. As shown in Fig 5, DDR48-heat shock protein and 12 kD heat shock protein expression drastically increased in the stipe tissue at growth stage 3. Additionally, under blue light conditions, the expression of conserved fungal protein initially increased in stipe tissue samples, but then decreased in these samples at growth stage 3. Sakamoto et al. [44] eported that DDR48-heat shock protein and conserved fungal protein were abundantly expressed in harvested *L*. *edodes* samples, whereas 12 kD heat shock protein was not. These differing results may be due to different cultivation conditions (25°C without light) than the conditions used in the present study. Nevertheless, in the present study, DDR48-heat shock protein, 12 kD heat shock protein, and conserved fungal protein were identified and highly expressed following blue light exposure.

Fasciclin-domain-containing protein and carbohydrate esterase family 4 protein are important to cell wall structure [45]. Additionally, fasciclin or fasciclin domain are involved in cell adhesion and affect the structure of cellulosic and non-cellulosic cell walls [46]. During fruit body development, genes important to cell wall structure may be more actively expressed in the mushroom tissue [47]. That is one of important role of cell wall structure genes in the fruit body development. Moreover, fasciclin-domain-containing protein and carbohydrate esterase family 4 protein were detected at similar expression levels in the fruit bodies of harvested mushroom [44]. These studies are in accordance with our own results in that fasciclin-domain-containing protein and carbohydrate esterase family 4 protein were constantly expressed in both the pileus and stipe of *L*. *edodes*. Despite a lack of research on f1 ATPase assembly protein 11 and alcohol oxidase-like protein in mushrooms, the present study found that these proteins were related to cell metabolism in *L*. *edodes*. Additionally, transcripts of these two proteins showed ambiguous expression in *L*. *edodes* in previous research [44].

FAD NAD-binding domain-containing protein is an important oxidoreductase with a role in electron transfer [48]. Because of its oxidoreductase activity, FAD NAD-binding domain-containing protein was classified as a photoreceptor in *L*. *edodes* [47]. As blue light was considered a main signal which prompts fruit body and pigment development [20,21], upregulated expression of FAD NAD-binding domain-containing protein would be a remarkable finding. In Fig 5, the expression level of FAD NAD-binding domain-containing protein under dark cultivation conditions was lower in the pileus and stipe tissue samples at growth stage 3 compared to samples exposed to blue light. Several researchers reported the relationship between the FAD binding gene and blue light in mushrooms. For example, Fu et al. [49] identified the FAD binding domain as a candidate gene in photomorphogenesis by blue light absorption. FAD-binding genes were further involved in the regulation of mycelial browning under blue light cultivation of *L*. *edodes* [47]. Therefore, we suggested that the FAD NAD-binding domain-containing protein encoding gene observed in this study might be involved in *L*. *edodes* photomorphogenesis induced by blue light.

Regarding downregulated expressed DEGs, it was reported that barwin-like endoglucanase was involved in polysaccharide metabolism and transport and that in mushrooms, these proteins were closely related to plant expansins [50]. As reported by Sakamoto et al. [44], barwin-like endoglucanase showed increased expression in the stipe tissue at growth stage 3 in harvested mushroom samples, in accordance with the results of this study (Fig 6). Similarly, the same study reported high expression of family S53 protease in harvested mushroom samples [44]; in the present study, family S53 protease showed higher expression levels in the stipe than in the pileus. Alternatively, we detected hydrophobin2 transcripts in both the pileus and the stipe at all growth stages (Fig 6). Ng et al. [51] identified hydrophobin genes from *L*. *edodes* and demonstrated that *Le*.*hyd2* was highly expressed in mycelial tissues. In addition, transcriptome analysis of an *L*. *edodes* fruit body by Sakamoto et al. [44] further corresponded to our results, high expression in fresh fruit body. Although a previous study reported that hydrophobin genes, such as *SC1* and *SC3*, were upregulated and involved in fruit body development under blue light or partial blue light in transcriptome analysis [47], the RT-PCR in this study demonstrated that the hydrophobin2 gene from *L*. *edodes* was dominantly expressed under dark cultivation conditions (Fig 6). Therefore, hydrophobin2 may be involved in hydrophobic coating during fruit body development under dark conditions.

During RNAseq analysis, 4 cytochrome P450 genes were screened as downregulated DEGs and we identified 2 different expression patterns during RT-PCR validation (Fig 6). However, the expression profiles with the primer sets, which were designed at conserved regions of 4 cytochrome P450, showed a similar expression pattern with Fig 6. Although the DEGs were selected for downregulation under blue light, the expression of cytochrome P450 generally increased with fruit body growth. Hsu et al. [52] reported strong expression of 10 cytochrome P450 genes from mushrooms during formation of the fruit body. Generally, cytochrome P450 functions as terminal oxidase enzymes in the electron transfer chain [53]. Therefore, cytochrome P450 may potentially be involved in light sensing termination or browning during mushroom fruit body development.

In this study, we identified candidate genes which were involved in various biological processes in *L*. *edodes* under blue light and dark conditions using RNAseq and validation with RT-PCR analyses during different stages of fruit body development. *L*. *edodes* cultivation under blue light induced enhanced pileus growth. Additionally, 12 candidate genes, including FAD NAD-binding domain-containing protein, were identified as up- or down-regulated genes under blue light cultivation. These results provide important information to further our understanding of blue light sensing and receptor mechanisms in the economically important mushroom species, *L*. *edodes*.

## Supporting information

S1 Table The list and annotation of 2-fold upregulated genes under blue light condition. (DOCX)

S2 Table The list of 2-fold upregulated genes selected by gene ontology enrichment analysis. (TXT)

S3 Table The list of 2-fold upregulated genes selected by KOG functional analysis. (TXT)

S4 Table The list of DEG primers with 4-fold change under blue light for qRT-PCR. (XLSX)

S5 Table The list of the transcriptome annotation and gene expression data. (XLSX)

S6 Table Effect of blue light on the properties of fruit body. (DOCX)

S1 Fig Morphological properties of *Lentinula edodes* grown under blue light or dark cultivation conditions. (TIF)
