# Supplementary material for: Transcriptome analysis of the edible mushroom Lentinula edodes in response to blue light
Source: PLoS One. 2020 Mar 27;15(3):e0230680. doi: 10.1371/journal.pone.0230680 (PMC7100940; doi:10.1371/journal.pone.0230680)
Supplement: S1 Table — (DOCX) [file pone.0230680.s001.docx]

| Gene ID | Fold change | p-value | Annotation | identity (%) | e-value |
| --- | --- | --- | --- | --- | --- |
| LENED_000001 | 2.560102356 | 0.021554789 | cytochrome p450 | 100 | 0 |
| LENED_000002 | 2.560102356 | 0.021554789 | cytochrome p450 | 100 | 0 |
| LENED_000038 | 2.594843859 | 0.013204771 | Transcriptional repressor XBP1 | 100 | 0 |
| LENED_000039 | 2.594843859 | 0.013204771 | Transcriptional repressor XBP1 | 100 | 0 |
| LENED_000082 | 2.879882721 | 0.020171923 | hypothetical protein LENED_000082 | 100 | 2E-101 |
| LENED_000141 | 2.032225966 | 0.005972322 | hypothetical protein LENED_000141 | 100 | 0 |
| LENED_000142 | 2.032225966 | 0.005972322 | hypothetical protein LENED_000142 | 100 | 0 |
| LENED_000179 | 3.214143379 | 0.009890819 | pleiotropic drug resistance ABC transporter | 100 | 0 |
| LENED_000180 | 3.214143379 | 0.009890819 | pleiotropic drug resistance ABC transporter | 100 | 0 |
| LENED_000286 | 6.102395699 | 0.012297812 | hypothetical protein LENED_000286 | 100 | 9E-134 |
| LENED_000366 | 5.08143098 | 0.000333737 | deoxyribodipyrimidine photo-lyase | 100 | 0 |
| LENED_000471 | 5.16257491 | 0.003973852 | halogenase | 100 | 0 |
| LENED_000490 | 2.327483083 | 0.016867064 | hypothetical protein LENED_000490 | 100 | 0 |
| LENED_000515 | 6.784577593 | 0.012371169 | hypothetical protein LENED_000515 | 100 | 0 |
| LENED_000529 | 2.094834169 | 0.003712296 | hypothetical protein LENED_000529 | 100 | 3E-49 |
| LENED_000593 | 2.033184309 | 0.000521175 | protein | 100 | 0 |
| LENED_000721 | 2.236071076 | 0.005685331 | caleosin domain containing protein | 100 | 0 |
| LENED_000888 | 2.429711862 | 0.015720317 | protein | 100 | 0 |
| LENED_000889 | 2.429711862 | 0.015720317 | protein | 100 | 0 |
| LENED_001069 | 2.381305044 | 0.025162569 | hypothetical protein LENED_001069 | 100 | 5E-168 |
| LENED_001226 | 23.95760079 | 0.032828478 | hypothetical protein LENED_001226 | 100 | 5E-26 |
| LENED_001227 | 20.50268937 | 0.002336096 | lea domain-containing protein | 100 | 3E-107 |
| LENED_001256 | 2.032220443 | 0.001023225 | major facilitator superfamily transporter domain-containing protein 5 | 100 | 5E-42 |
| LENED_001349 | 3.078262612 | 0.00537211 | extracellular triacylglycerol lipase precursor | 100 | 0 |
| LENED_001350 | 3.078262612 | 0.00537211 | extracellular triacylglycerol lipase precursor | 100 | 0 |
| LENED_001351 | 3.078262612 | 0.00537211 | extracellular triacylglycerol lipase precursor | 100 | 0 |
| LENED_001445 | 10.85500292 | 0.032182616 | hypothetical protein LENED_001445 | 100 | 5E-101 |
| LENED_001494 | 2.075271679 | 4.3993E-06 | hypothetical protein LENED_001494 | 100 | 0 |
| LENED_001549 | 2.738584959 | 0.002093756 | cytochrome P450 | 100 | 0 |
| LENED_001557 | 2.134784838 | 0.004603165 | hypothetical protein LENED_001557 | 100 | 0 |
| LENED_001611 | 7.205691408 | 0.000893853 | FAD NAD -binding domain-containing protein | 100 | 0 |
| LENED_001617 | 2.30966574 | 0.035419856 | carbohydrate esterase family 8 protein | 100 | 0 |
| LENED_001618 | 2.30966574 | 0.035419856 | carbohydrate esterase family 8 protein | 100 | 0 |
| LENED_001673 | 2.031278494 | 0.003275702 | S-adenosyl-L-methionine-dependent methyltransferase | 100 | 0 |
| LENED_001775 | 2.291690948 | 0.02813758 | Acireductone dioxygenase | 100 | 0 |
| LENED_001823 | 2.250302668 | 0.027972174 | short-chain dehydrogenase reductase sdr | 100 | 8E-177 |
| LENED_001825 | 3.591903801 | 0.020998239 | alpha beta-hydrolase | 100 | 0 |
| LENED_001863 | 2.828978668 | 0.003281995 | gcn5-related n-acetyltransferase family protein | 100 | 9E-136 |
| LENED_001875 | 13.24946562 | 0.003817869 | hypothetical protein LENED_001875 | 100 | 7E-98 |
| LENED_002175 | 2.115316782 | 0.043296839 | ribosomal protein L4 | 100 | 1E-146 |
| LENED_002521 | 2.107621744 | 0.001336087 | glycoside hydrolase family 71 protein | 100 | 0 |
| LENED_002522 | 2.107621744 | 0.001336087 | glycoside hydrolase family 71 protein | 100 | 0 |
| LENED_002627 | 2.252561949 | 0.008160225 | ubiquitinyl hydrolase | 100 | 0 |
| LENED_002628 | 2.252561949 | 0.008160225 | ubiquitinyl hydrolase | 100 | 0 |
| LENED_002635 | 2.531222297 | 0.028809006 | anaphase-promoting complex subunit 2 | 100 | 0 |
| LENED_002791 | 2.731376191 | 0.002230417 | hypothetical protein LENED_002791 | 100 | 5E-60 |
| LENED_002792 | 2.686701399 | 0.004352471 | hypothetical protein LENED_002792 | 100 | 2E-46 |
| LENED_002810 | 18.06196717 | 0.00016074 | heat shock protein 9 | 100 | 9E-43 |
| LENED_002835 | 3.188945493 | 0.009268966 | DUF1264-domain-containing protein | 100 | 3E-174 |
| LENED_002897 | 4.710687108 | 0.014581111 | hypothetical protein LENED_002897 | 100 | 2E-09 |
| LENED_002918 | 2.118171759 | 0.012648747 | hypothetical protein LENED_002918 | 100 | 1E-159 |
| LENED_002949 | 3.472616744 | 0.014856047 | hypothetical protein LENED_002949 | 100 | 6E-117 |
| LENED_003093 | 14.95137756 | 0.031694057 | hypothetical protein LENED_003093 | 100 | 5E-65 |
| LENED_003136 | 2.071790336 | 0.005599655 | hypothetical protein LENED_003136 | 100 | 2E-41 |
| LENED_003208 | 2.09159913 | 0.023246578 | rad26-like snf2 family dna-dependent atpase | 100 | 0 |
| LENED_003209 | 2.09159913 | 0.023246578 | rad26-like snf2 family dna-dependent atpase | 100 | 0 |
| LENED_003210 | 2.09159913 | 0.023246578 | rad26-like snf2 family dna-dependent atpase | 100 | 0 |
| LENED_003243 | 25.70503616 | 0.029143739 | mismatched base pair and cruciform DNA recognition protein | 100 | 5E-70 |
| LENED_003330 | 2.204841125 | 0.002603535 | 3-deoxy-7-phosphoheptulonate synthase | 100 | 0 |
| LENED_003425 | 4.652105607 | 0.002948353 | hypothetical protein LENED_003425 | 100 | 8E-139 |
| LENED_003427 | 2.316924872 | 0.00944225 | P-loop containing nucleoside triphosphate hydrolase protein | 100 | 0 |
| LENED_003428 | 2.316924872 | 0.00944225 | P-loop containing nucleoside triphosphate hydrolase protein | 100 | 0 |
| LENED_003634 | 3.862874422 | 0.016092793 | hypothetical protein LENED_003634 | 100 | 4E-87 |
| LENED_003779 | 2.180158372 | 0.017235375 | signal transducer | 100 | 0 |
| LENED_003780 | 2.180158372 | 0.017235375 | signal transducer | 100 | 0 |
| LENED_003869 | 2.151941481 | 0.009677828 | MFS general substrate transporter | 100 | 0 |
| LENED_004060 | 2.117014473 | 0.009497741 | hypothetical protein LENED_004060 | 100 | 0 |
| LENED_004061 | 2.117014473 | 0.009497741 | hypothetical protein LENED_004061 | 100 | 0 |
| LENED_004065 | 2.362635776 | 0.034962781 | hypothetical protein LENED_004065 | 100 | 0 |
| LENED_004066 | 2.362635776 | 0.034962781 | cell cycle control protein | 100 | 0 |
| LENED_004249 | 2.776167011 | 0.02472865 | ctg1 protein | 100 | 1E-76 |
| LENED_004250 | 2.776167011 | 0.02472865 | ctg1 protein | 100 | 1E-82 |
| LENED_004342 | 2.423401944 | 0.004804268 | phosphatidylserine synthase 2 | 100 | 0 |
| LENED_004343 | 2.423401944 | 0.004804268 | phosphatidylserine synthase 2 | 100 | 0 |
| LENED_004394 | 2.436734543 | 0.019880692 | hypothetical protein LENED_004394 | 100 | 3E-74 |
| LENED_004424 | 2.871229388 | 0.039645332 | carbohydrate-binding module family 13 protein | 100 | 8E-100 |
| LENED_004547 | 2.256527137 | 0.011697234 | nadp-specific glutamate dehydrogenase | 100 | 0 |
| LENED_004554 | 4.920377538 | 0.000493162 | carbohydrate esterase family 4 protein | 100 | 0 |
| LENED_004582 | 3.037723857 | 0.001650322 | alpha-amylase | 100 | 0 |
| LENED_004676 | 2.161801131 | 0.039748252 | glycoside hydrolase family 28 protein | 100 | 0 |
| LENED_004693 | 11.403711 | 0.000736095 | hypothetical protein LENED_004693 | 100 | 0 |
| LENED_004694 | 11.403711 | 0.000736095 | hypothetical protein LENED_004694 | 100 | 0 |
| LENED_004696 | 2.538098741 | 0.000506575 | mannitol-1-phosphate dehydrogenase | 100 | 0 |
| LENED_004700 | 7.034460549 | 0.012197607 | glycoside hydrolase family 3 protein | 100 | 0 |
| LENED_004701 | 7.034460549 | 0.012197607 | glycoside hydrolase family 3 protein | 100 | 0 |
| LENED_004807 | 3.231327685 | 0.00502042 | hypothetical protein LENED_004807 | 100 | 4E-18 |
| LENED_005077 | 2.237870685 | 0.010014608 | aquaporin 4 | 100 | 0 |
| LENED_005078 | 2.581585675 | 0.006775056 | aquaporin 4 | 100 | 0 |
| LENED_005174 | 4.559023009 | 0.022340459 | hypothetical protein LENED_005174 | 100 | 1E-120 |
| LENED_005175 | 4.559023009 | 0.022340459 | hypothetical protein LENED_005175 | 100 | 7E-120 |
| LENED_005193 | 22.57803737 | 0.003785848 | hypothetical protein LENED_005193 | 100 | 4E-84 |
| LENED_005208 | 2.195326742 | 0.045653068 | hypothetical protein LENED_005208 | 100 | 0 |
| LENED_005495 | 3.352665342 | 0.000865479 | Esterase lipase thioesterase | 100 | 2E-159 |
| LENED_005496 | 4.078681562 | 0.000794942 | hypothetical protein LENED_005496 | 100 | 3E-49 |
| LENED_005526 | 2.082553259 | 0.015070873 | phosphoribosylglycinamide synthetase | 100 | 0 |
| LENED_005527 | 2.082553259 | 0.015070873 | Zn -C6 fungal-type transcription factor | 100 | 0 |
| LENED_005700 | 2.429711862 | 0.015720317 | hypothetical protein LENED_005700 | 100 | 1E-148 |
| LENED_005737 | 2.666964691 | 0.00789193 | Isochorismatase hydrolase | 100 | 0 |
| LENED_006238 | 2.455707964 | 0.006969053 | erg4 erg24 ergosterol biosynthesis-like protein | 100 | 5E-163 |
| LENED_006379 | 3.017234338 | 0.03572336 | oxidase-like protein | 100 | 0 |
| LENED_006380 | 2.781339393 | 0.023713746 | oxidase-like protein | 100 | 0 |
| LENED_006419 | 5.335978179 | 0.003645015 | Di-copper centre-containing protein | 100 | 0 |
| LENED_006620 | 8.431318847 | 0.000391298 | serine protease inhibitor | 100 | 7E-102 |
| LENED_006667 | 2.150609696 | 0.045073443 | P-loop containing nucleoside triphosphate hydrolase protein | 100 | 0 |
| LENED_006721 | 4.046883779 | 0.03188562 | hypothetical protein LENED_006721 | 100 | 2E-152 |
| LENED_006745 | 4.588641333 | 0.033543532 | hypothetical protein LENED_006745 | 100 | 3E-15 |
| LENED_006787 | 5.789180719 | 0.01112576 | lea domain protein | 100 | 5E-117 |
| LENED_006823 | 2.459750379 | 0.007028883 | mnn4-regulates the mannosylphosphorylation | 100 | 0 |
| LENED_006895 | 2.498416063 | 0.0087819 | lea domain-containing protein | 100 | 4E-143 |
| LENED_006902 | 6.688643083 | 0.019981646 | glycosyl transferase family 2 protein | 100 | 1E-10 |
| LENED_006924 | 3.735265649 | 0.000898578 | O-methylsterigmatocystin oxidoreductase | 100 | 0 |
| LENED_006935 | 2.429711862 | 0.015720317 | multidrug resistance protein cdr1 | 100 | 0 |
| LENED_006942 | 6.924732319 | 6.48567E-05 | AChain Crystal Structure Of Glutathione Transferase Gstfua3 From Phanerochaete Chrysosporium | 100 | 2E-82 |
| LENED_006959 | 2.05357054 | 0.004253416 | hypothetical protein LENED_006959 | 100 | 0 |
| LENED_006960 | 2.05357054 | 0.004253416 | hypothetical protein LENED_006960 | 100 | 0 |
| LENED_006961 | 2.05357054 | 0.004253416 | hypothetical protein LENED_006961 | 100 | 0 |
| LENED_006990 | 4.155846242 | 0.001001477 | alpha beta-hydrolase | 100 | 0 |
| LENED_006994 | 2.242344996 | 0.021483613 | WD40 repeat-like protein | 100 | 0 |
| LENED_007189 | 2.672805705 | 0.008813773 | atp-dependent protease | 100 | 2E-160 |
| LENED_007190 | 2.672805705 | 0.008813773 | atp-dependent protease | 100 | 0 |
| LENED_007342 | 7.454419382 | 2.92023E-06 | hypothetical protein LENED_007342 | 100 | 2E-129 |
| LENED_007359 | 2.126213286 | 0.017826565 | membrane protein | 100 | 5E-173 |
| LENED_007487 | 2.205182584 | 0.023838265 | hypothetical protein LENED_007487 | 100 | 2E-168 |
| LENED_007557 | 2.718350344 | 0.0011719 | related to MSS116-RNA helicase of the DEAD box mitochondrial | 100 | 0 |
| LENED_007705 | 2.884014959 | 0.010150668 | hypothetical protein LENED_007705 | 100 | 0 |
| LENED_007757 | 9.889104784 | 0.015547895 | hypothetical protein LENED_007757 | 100 | 0 |
| LENED_007821 | 3.088701421 | 0.036459992 | hypothetical protein LENED_007821 | 100 | 5E-97 |
| LENED_007961 | 2.031530352 | 0.015402007 | helix-turn-helix transcription type | 100 | 8E-81 |
| LENED_008199 | 2.05400428 | 0.017370348 | MFS general substrate transporter | 100 | 0 |
| LENED_008200 | 2.05400428 | 0.017370348 | MFS general substrate transporter | 100 | 0 |
| LENED_008225 | 2.536779283 | 0.036613118 | quinate permease | 100 | 0 |
| LENED_008284 | 3.123142577 | 0.003683438 | hypothetical protein LENED_008284 | 100 | 3E-67 |
| LENED_008302 | 2.409034184 | 0.024444753 | hypothetical protein LENED_008302 | 100 | 3E-43 |
| LENED_008303 | 2.409034184 | 0.024444753 | hypothetical protein LENED_008303 | 100 | 2E-90 |
| LENED_008422 | 2.181430479 | 0.009171532 | Dual specificity phosphatase ibp1 | 100 | 0 |
| LENED_008423 | 2.181430479 | 0.009171532 | Dual specificity phosphatase ibp1 | 100 | 0 |
| LENED_008467 | 2.78239147 | 0.034455732 | carbohydrate-binding module family 21 protein | 100 | 0 |
| LENED_008468 | 3.736720297 | 0.038065942 | carbohydrate-binding module family 21 protein | 100 | 9E-145 |
| LENED_008539 | 2.119319324 | 0.017957471 | FAD NAD-P-binding domain-containing protein | 100 | 0 |
| LENED_008756 | 5.039430199 | 0.013836475 | hypothetical protein LENED_008756 | 100 | 0 |
| LENED_008757 | 5.039430199 | 0.013836475 | hypothetical protein LENED_008757 | 100 | 0 |
| LENED_008877 | 2.013518334 | 0.014072511 | protein | 100 | 0 |
| LENED_008933 | 3.358599198 | 0.016677014 | hypothetical protein LENED_008933 | 100 | 6E-67 |
| LENED_008935 | 6.005975968 | 0.000485663 | alpha beta hydrolase fold protein | 100 | 0 |
| LENED_009062 | 4.478871487 | 0.006947914 | NAD -binding protein | 100 | 0 |
| LENED_009231 | 2.934549962 | 0.002334549 | thaumatin-like protein | 100 | 0 |
| LENED_009241 | 2.240279464 | 0.021402027 | hypothetical protein LENED_009241 | 100 | 6E-69 |
| LENED_009250 | 2.110904856 | 0.009318243 | expansin family protein | 100 | 3E-69 |
| LENED_009313 | 12.56184904 | 0.000484101 | conserved fungal protein | 100 | 0 |
| LENED_009326 | 11.13373573 | 0.036367665 | beta-glucosidase 1 precursor | 100 | 0 |
| LENED_009327 | 2.402744195 | 0.010158154 | hypothetical protein LENED_009327 | 100 | 0 |
| LENED_009495 | 2.004674972 | 0.027248083 | alkaline phosphatase family protein | 100 | 4E-152 |
| LENED_009517 | 6.408421634 | 0.000131464 | Fasciclin-domain-containing protein | 100 | 2E-166 |
| LENED_009532 | 2.540498091 | 0.001419614 | glycoside hydrolase family 128 protein | 100 | 0 |
| LENED_009533 | 2.540498091 | 0.001419614 | glycoside hydrolase family 128 protein | 100 | 0 |
| LENED_009535 | 3.261550561 | 0.001246757 | hypothetical protein LENED_009535 | 100 | 1E-99 |
| LENED_009536 | 3.779490535 | 0.000804852 | family A1 protease | 100 | 0 |
| LENED_009572 | 4.9048063 | 0.005829363 | hypothetical protein LENED_009572 | 100 | 5E-36 |
| LENED_009740 | 2.280531562 | 0.04250563 | FAD NAD-P-binding domain-containing protein | 100 | 0 |
| LENED_009785 | 2.55793836 | 0.006245696 | terpenoid synthase | 100 | 0 |
| LENED_009912 | 2.504478476 | 0.006768136 | geranylgeranyl transferase type-1 subunit beta | 100 | 0 |
| LENED_009977 | 5.101528489 | 0.001272231 | hypothetical protein LENED_009977 | 100 | 0 |
| LENED_009978 | 5.101528489 | 0.001272231 | hypothetical protein LENED_009978 | 100 | 0 |
| LENED_009984 | 29.22159838 | 0.029928801 | related to DDR48-Heat shock protein | 100 | 2E-94 |
| LENED_009985 | 6.433728792 | 0.005375578 | related to DDR48-Heat shock protein | 100 | 6E-93 |
| LENED_010060 | 2.802210191 | 0.021072568 | hypothetical protein LENED_010060 | 100 | 9E-144 |
| LENED_010077 | 2.401098772 | 0.002512579 | uv-damage endonuclease | 100 | 0 |
| LENED_010130 | 2.116117131 | 0.021162499 | salicylate hydroxylase | 100 | 0 |
| LENED_010310 | 7.448166537 | 0.037663511 | hypothetical protein LENED_010310 | 100 | 0 |
| LENED_010311 | 7.448166537 | 0.037663511 | hypothetical protein LENED_010311 | 100 | 0 |
| LENED_010370 | 2.097079347 | 0.010491843 | lea domain-containing protein | 100 | 1E-118 |
| LENED_010402 | 4.298776233 | 0.024190655 | f1 atpase assembly protein 11 | 100 | 1E-65 |
| LENED_010485 | 8.184082222 | 0.017388109 | hypothetical protein LENED_010485 | 100 | 8E-92 |
| LENED_010490 | 4.045960112 | 0.00491688 | fumarylacetoacetate hydrolase | 100 | 0 |
| LENED_010625 | 2.536779283 | 0.036613118 | glycoside hydrolase family 5 protein | 100 | 0 |
| LENED_010626 | 2.536779283 | 0.036613118 | glycoside hydrolase family 5 protein | 100 | 0 |
| LENED_010652 | 19.16279944 | 0.026373622 | alpha beta-hydrolase | 100 | 0 |
| LENED_010653 | 19.16279944 | 0.026373622 | alpha beta-hydrolase | 100 | 0 |
| LENED_010751 | 2.005614453 | 0.014310194 | hypothetical protein LENED_010751 | 100 | 0 |
| LENED_010795 | 5.884683086 | 0.000400499 | hypothetical protein LENED_010795 | 100 | 6E-125 |
| LENED_010858 | 26.88011126 | 0.01090325 | 12 kda heat shock protein (glucose and lipid-regulated protein) | 100 | 2E-50 |
| LENED_010917 | 2.417811922 | 0.009603837 | hypothetical protein LENED_010917 | 100 | 0 |
| LENED_010918 | 2.417811922 | 0.009603837 | hypothetical protein LENED_010918 | 100 | 4E-61 |
| LENED_010983 | 2.101805235 | 0.000615772 | indole-diterpene biosynthesis protein | 100 | 0 |
| LENED_011077 | 3.050280177 | 0.041897476 | hypothetical protein LENED_011077 | 100 | 0 |
| LENED_011078 | 3.49007768 | 0.034629278 | hypothetical protein LENED_011078 | 100 | 0 |
| LENED_011168 | 4.181073546 | 0.03639206 | alcohol oxidase-like protein | 100 | 0 |
| LENED_011280 | 2.873608596 | 0.040307766 | plant expansin | 100 | 3E-82 |
| LENED_011381 | 2.772472059 | 0.004905333 | transmembrane gtpase fzo1 | 100 | 0 |
| LENED_011382 | 2.018945581 | 0.046505664 | transmembrane gtpase fzo1 | 100 | 0 |
| LENED_011406 | 2.042139192 | 0.010918247 | u3 snornp-associated protein cic1 utp30 family protein | 100 | 0 |
| LENED_011407 | 2.042139192 | 0.010918247 | u3 snornp-associated protein cic1 utp30 family protein | 100 | 0 |
| LENED_011421 | 30.07615712 | 1.94926E-05 | mismatched base pair and cruciform DNA recognition protein | 100 | 4E-58 |
| LENED_011432 | 23.00446146 | 0.006825335 | hypothetical protein LENED_011432 | 100 | 8E-55 |
| LENED_011578 | 2.417811922 | 0.009603837 | cytochrome p450 | 100 | 0 |
| LENED_011579 | 2.417811922 | 0.009603837 | cytochrome p450 | 100 | 0 |
| LENED_011587 | 2.263584545 | 0.015978815 | cytochrome p450 | 100 | 0 |
| LENED_011588 | 2.263584545 | 0.015978815 | cytochrome p450 | 100 | 0 |
| LENED_011621 | 3.590938043 | 0.000839162 | Meiotically up-regulated gene 180 protein | 100 | 0 |
| LENED_011622 | 2.428726513 | 0.015680525 | alpha beta hydrolase fold protein | 100 | 0 |
| LENED_011692 | 4.277534379 | 0.001495089 | hypothetical protein LENED_011692 | 100 | 9E-32 |
| LENED_011887 | 2.036045877 | 0.026902903 | C2H2 type zinc finger domain-containing protein | 100 | 0 |
| LENED_011925 | 3.260861375 | 0.006010206 | glycoside hydrolase family 30 protein | 100 | 0 |
| LENED_011938 | 2.278777822 | 0.02458887 | hypothetical protein LENED_011938 | 100 | 0 |
| LENED_012091 | 3.306967813 | 0.018809614 | pre-mRNA-splicing factor ATP-dependent RNA helicase PRP16 | 100 | 0 |
| LENED_012158 | 2.075271679 | 4.3993E-06 | hypothetical protein LENED_012158 | 100 | 1E-99 |
| LENED_012159 | 2.075271679 | 4.3993E-06 | hypothetical protein LENED_012159 | 100 | 1E-101 |
| LENED_012320 | 3.611864379 | 0.002100235 | salicylate hydroxylase | 100 | 0 |
| LENED_012321 | 3.611864379 | 0.002100235 | salicylate hydroxylase | 100 | 0 |
| LENED_012330 | 2.451692337 | 0.036099616 | hypothetical protein LENED_012330 | 100 | 5E-77 |
| LENED_012543 | 6.729582011 | 0.024958028 | proteophosphoglycan ppg4 | 100 | 6E-86 |
| LENED_012567 | 2.37840946 | 0.00785905 | other 1 protein kinase | 100 | 0 |
| LENED_012636 | 4.259074547 | 0.010195774 | hypothetical protein LENED_012636 | 100 | 7E-78 |
| LENED_012703 | 6.764324333 | 0.024383309 | hypothetical protein LENED_012703 | 100 | 1E-54 |
| LENED_012776 | 2.12209533 | 0.018942465 | eukaryotic initiation factor 4f subunit p130 | 100 | 7E-67 |
| LENED_012782 | 2.128752228 | 0.027830996 | hypothetical protein LENED_012782 | 100 | 0 |
| LENED_012802 | 2.592521892 | 0.019452474 | hypothetical protein LENED_012802 | 100 | 0 |
| LENED_012831 | 2.431867124 | 0.032064254 | U1 snRNP 70K protein | 100 | 4E-175 |
| LENED_012832 | 2.431867124 | 0.032064254 | U1 snRNP 70K protein | 100 | 0 |
| LENED_012860 | 2.440304957 | 0.029219667 | hypothetical protein LENED_012860 | 100 | 3E-105 |
| LENED_012861 | 2.440304957 | 0.029219667 | hypothetical protein LENED_012861 | 100 | 6E-129 |
