# Supplementary material for: Transcriptome analysis of the edible mushroom Lentinula edodes in response to blue light
Source: PLoS One. 2020 Mar 27;15(3):e0230680. doi: 10.1371/journal.pone.0230680 (PMC7100940; doi:10.1371/journal.pone.0230680)
Supplement: S6 Table — (DOCX) [file pone.0230680.s006.docx]

| Diameter of pileus | | | | Thickness of pileus | | | | Length os stipe | | | | Diameter of stipe | | | |
| --- | --- | --- | --- | --- | --- | --- | --- | --- | --- | --- | --- | --- | --- | --- | --- |
| stage | light condition | | t-test | stage | light condition | | t-test | stage | light condition | | t-test | stage | light condition | | t-test |
|  | blue | dark |  |  | blue | dark |  |  | blue | dark |  |  | blue | dark |  |
| 1 | 18.8 | 13.9 | ** | 1 | 11.9 | 7.8 | * | 1 | 13.1 | 23.4 | ** | 1 | 12.2 | 12.4 |  |
| 2 | 38.9 | 17.0 | ** | 2 | 15.7 | 12.3 |  | 2 | 27.9 | 34.6 | ** | 2 | 16.3 | 17.4 |  |
| 3 | 45.2 | 27.4 | *** | 3 | 25.9 | 23.9 |  | 3 | 41.7 | 61.1 | *** | 3 | 30.5 | 33.2 |  |

Asterisks mean significantly difference at p < 0.05, 0.01, and 0.0001 according to Student’s *t*-test.

* represents p < 0.05, ** represents p < 0.01, *** represents p < 0.001.
