# Supplementary figures and images for: Transcriptome analysis of the edible mushroom Lentinula edodes in response to blue light
Source: PLoS One. 2020 Mar 27;15(3):e0230680. doi: 10.1371/journal.pone.0230680 (PMC7100940; doi:10.1371/journal.pone.0230680)

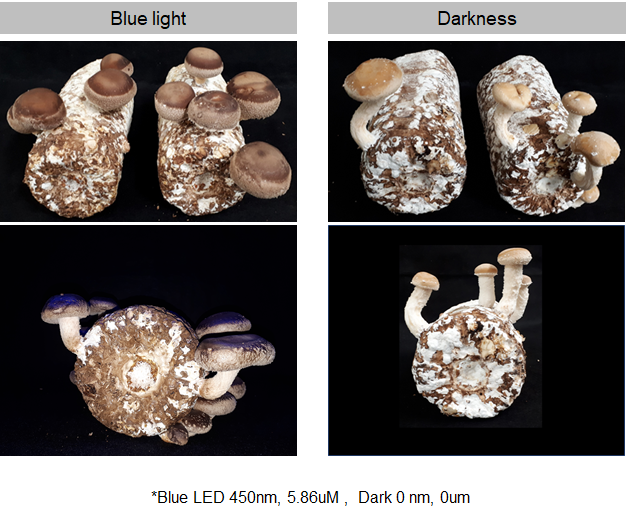

Supplement: S1 Fig — (TIF) [file pone.0230680.s007.tif]
